# PICU associated social-emotional adversity in early childhood cancer patients: consequences in neurodevelopment and mental health

**DOI:** 10.3389/fonc.2026.1627533

**Published:** 2026-02-13

**Authors:** Maria Kroupina, Quannah Parker-McGowan, Madeline Kotz, Arif Somani

**Affiliations:** 1 Department of Pediatrics, Division of Clinical Behavioral Neuroscience, University of Minnesota, Minneapolis, MN, United States; 2 Department of Pediatrics, Division of Pediatric Critical Care Medicine, University of Minnesota, Minneapolis, MN, United States

**Keywords:** early childhood cancer, mental health, neurodevelopment, PICU hospitalization, social-emotional adversity

## Abstract

Here we propose that PICU hospitalization accentuates extreme early social-emotional adversity and compounds hospital-associated trauma that may result in neurodevelopmental and mental health challenges for pediatric cancer survivors. We focus specifically on the first three years of life, when the incidence of childhood cancer is the highest and the brain is in its most vulnerable period of development. A comprehensive understanding of risks associated with PICU hospitalization is necessary for informing improvements to the inpatient environment and proposing new models for administering mental health care, evidence-based intervention, and long-term follow-up.

## Defining the problem

Each year, approximately 15,000 children and adolescents in the United States are diagnosed with cancer. From 2003-2019, the incidence was highest among infants (264.6/1,000,000) and young children (up to age 5: 230.7/1,000,000), with this group making up almost one third of childhood cancer diagnoses (1). Fortunately, pediatric cancer survival in the United States has increased greatly since the 1970s, and continues to improve, from 82% survival in 2001–2007 to 85.1% in 2008-2015 (2). Accordingly, the number of pediatric cancer survivors is growing, estimated at 483,039 individuals in the United States in 2018 (1). This has resulted in increased focus on long-term outcomes and late effects of treatment in pediatric cancer survivors, as well as efforts to improve longitudinal follow-up and care.

Limited literature exists to describe neurodevelopmental outcomes in patients receiving cancer diagnosis and treatment in early childhood, but in a study of 29 children under 3 years, 82.6% were found to have below average cognitive functioning and 69.3% had below average adaptive functioning (3). In samples that encompass survivors across age groups, neurodevelopmental challenges are present in one third of pediatric cancer survivors, most commonly in the areas of visual processing, visual-motor functioning, attention, and executive functioning (4, 5). For mental health outcomes, there is similarly limited data describing the early childhood population, but psychosocial late effects, primarily post-traumatic stress disorder, anxiety, and depression; have been recognized in pediatric cancer survivors more broadly, particularly among those with additional risk factors, including more intensive treatment and poor family functioning (6).

Much of the existing literature evaluating these outcomes in pediatric cancer survivors attributes neurodevelopmental and mental health consequences to the impact of chemotherapy and other treatments on the developing brain (4, 5). More recently, it has been argued that pediatric cancer treatment be categorized as an adverse childhood experience; thereby, positing a direct relationship between treatment-related adversity and neurodevelopmental and mental health challenges in later life (7). An experience that encompasses much of this treatment-related adversity is hospitalization, which pediatric cancer patients often experience on a prolonged and repeated basis over their treatment course. Importantly, up to 38% require advanced care in the pediatric intensive care unit (PICU) (8, 9). Outcomes in PICU patients have been conceptualized by the Integrative Trajectory Model of Pediatric Medical Traumatic Stress and the PICS-p (Post-Intensive Care Syndrome in Pediatrics) framework (10–13). Both models conceptualize post-discharge effects on cognitive function and mental and social health imposed not only on the patient, but also parents and siblings. Across various illnesses, the incidence of clinical or subclinical post-medical traumatic stress is approximately 30% in both patients and parents; and may remain chronic and persistent (10). For instance, delirium in PICU patients is associated with post-discharge decline in health related quality of life, sleep disturbance and parental anxiety (14–16). In a prospective, sibling-matched study, PICU patients requiring invasive mechanical ventilation scored lower in IQ, visuospatial skills, non-verbal memory and fine motor control than their siblings (17). Given the very nature of critical and life saving care mandated by a tenuous clinical condition, PICU admission represents additional complexity and morbidity in the care of these young patients.

Here we propose that PICU hospitalization accentuates extreme early social-emotional adversity and compounds hospital-associated trauma that may result in neurodevelopmental and mental health challenges for pediatric cancer survivors. We focus specifically on the first three years of life, when the incidence of childhood cancer is the highest *and* the brain is in its most vulnerable period of development. A comprehensive understanding of risks associated with PICU hospitalization is necessary for informing improvements to the inpatient environment and proposing new models for administering mental health care, evidence-based intervention, and long-term follow-up.

## PICU hospitalization as a form of early social-emotional adversity

Hospitalization, especially in the PICU, often necessitates medical and surgical procedures, as well as ongoing, life-preserving modalities that in and of themselves introduce pain, discomfort, delirium and added morbidities (14–17). Further, primary caregivers are not always able to remain in the hospital with their child at all times. Other children in the home, employment constraints, transportation limitations, and financial burdens are examples of common obstacles that prevent caregivers from remaining at the bedside. Language, cultural, and even necessary medical devices and technology pose other barriers to the child-caregiver dyad, even when caregivers can be at the bedside. As such, these young patients are essentially alone for extended periods of time, with only nurses, physicians, and related service providers as their main sources of support (18, 19).

Complex medical illness and associated hospitalization (including the PICU admission) introduces stress to the patient; but, they also compound the risk of disrupting and impeding the child-parent relationship, which is fundamental to stress regulation in a child – the consequences of which may be particularly devastating for young children during this sensitive period of brain development (19, 20). The effects of PICU hospitalization on child-caregiver relationships lie on a spectrum. At the more severe end, these disruptions can include emotional isolation, impaired bonding, and even neglect. In such cases, the prolonged or intense stress may reach levels that constitute toxic stress, posing risks to the child’s long-term mental health and neurodevelopment. Toxic stress occurs when a young child has experiences which “produce frequent, strong, and/or prolonged activations of the body’s stress response systems in the absence of the protection of a supportive adult relationship”.^21(p32)^ Without intervention and caregiver support, toxic stress can negatively impact a child’s ability to regulate stress hormones, such as cortisol, as well as cause functional and structural changes to brain structures involved in stress regulation, including the amygdala and hippocampus (22, 23). Moreover, toxic stress puts a child’s health at risk and is linked to long-term health conditions including cardiovascular disease, diabetes, depression, and a weakened immune system (22). Stressors to the child also heighten caregiver anxiety and contribute to a deleterious feedback cycle in the child-caregiver dyad and family unit (24, 25).

A further consequence of hospitalization in the PICU for young pediatric cancer patients is the potential impediment on a child’s ability to explore and learn from their environment, which is crucial for helping children learn skills such as decision making (26). In addition to environmental obstacles (e.g. medical devices, monitors, and therapeutics) that negatively impact exploration, compromised early child-caregiver relationships can also fundamentally alter how a child learns and adapts. Specifically, young children will still approach a novel object, even in the face of aversive stimuli, if their caregivers are present (23, 27). Complex, chronic and repeated hospitalizations can disrupt typical developmental processes in young children, in part because of the unpredictable environment and stressors alluded to above (19). As an example of which, we presented a case study of a 21-month-old male diagnosed with neuroblastoma. Over the course of a nine-month hospitalization, this patient suffered expressive language regression and loss of effective communication skills (19).

It has been suggested that early brain plasticity cuts both ways. The developing brain is highly sensitive to both positive and negative experiences; thus, making early childhood a particularly vulnerable period of development (28–30). Research on the first 1000 days (from conception to age 2) references this concept and further adds that maternal mental health impacts parenting practices as well as fetal and infant growth and bonding (31). Indeed, it has been postulated that the fetal milieu and post natal care plays an underlying role in the development of chronic non-communicable diseases of adulthood such as cardiovascular diseases, type 2 diabetes mellitus, hypertension, and obesity (32, 33). This is further expanded upon in the next 1000 days (age 2 to 5) where holistic development may mitigate lost opportunities and optimize overall outcomes (34). In particular, the second 1000 days expands on cognitive, language and social emotional skills, including caregiver–child attachment security, each of which influence downstream play-based learning, acquisition of early literacy and numeracy skills, and school readiness; and ultimately, executive functioning (35). Investment in early childhood care and education may attenuate adolescent risk taking behavior, truancy, crime, welfare dependency, and improve labor outcomes. The potential return of investment is 8–19 times larger than the cost of implementing these programs across low and middle income countries (36).

**Figure 1:**
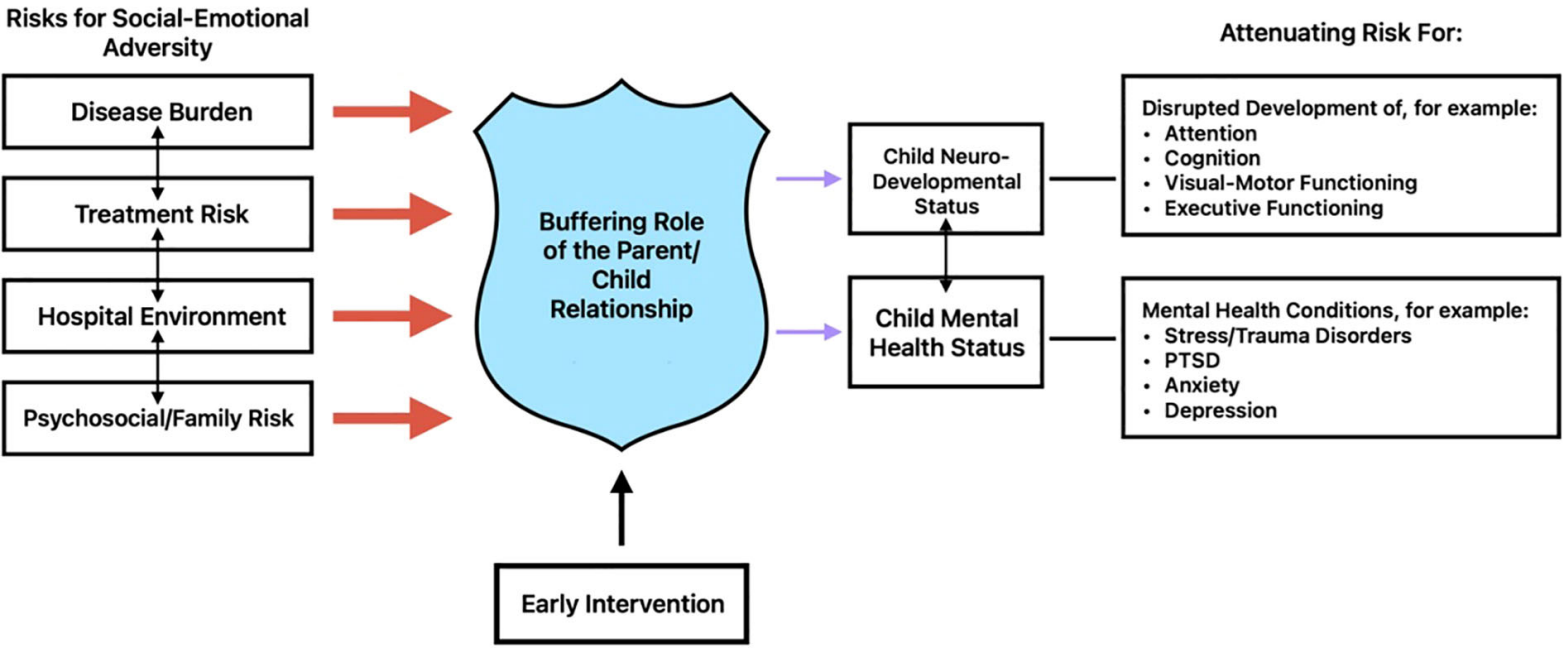
Early, targeted interventions accentuate the role of the parent-child relationship buffer, attenuating the impact of social-emotional adversity on downstream neurodeveloment and mental health outcomes.

Adverse conditions in early life, such as pediatric cancer and PICU hospitalization, put a child’s neurodevelopment and mental health at risk. On the other hand, early brain plasticity offers a unique window of opportunity for intervention. Because the brain is so adaptable during early childhood, timely early intervention can significantly improve outcomes and even reverse some of the negative impacts of early adversity. While it is likely that hospitalization in the PICU for young pediatric cancer patients can have negative impacts on their mental health and development, caregiver-child relationships can act as a “buffer” to toxic stress for medically complex children (21). Close emotional bonds with caregivers are an important protective factor for children (37, 38). Early intervention targeting child-caregiver relationships and optimizing co-regulation of stress have been found to have a long-lasting, positive impact on multiple domains, including stress regulation, emotional regulation, and more optimal neurodevelopmental functioning, such as executive functioning (39–42). In addition, child-caregiver dyadic interventions have been found to improve secure attachment and verbal abilities, as well as improve parent sensitivity (**Figure 1**) (43–45).

## Future directions

Given the extremely sensitive period of development and the added complexity that hospitalization can bring to pediatric cancer patients requiring PICU admission, the approach to assessment and intervention must be systematic and evidence-based in order to ensure optimal neurodevelopmental and mental health outcomes. These approaches must begin at the time of the child’s hospitalization and extend beyond discharge to support optimal developmental, cognitive, emotional, and psychosocial outcomes. A collaborative, team-based approach to assessment and intervention, including input from pediatric psychologists, inpatient social workers, physicians, and child life specialists, would facilitate such care delivery. In this collaborative and individually tailored approach, assessing the mental health needs of the child and their family, and soliciting their engagement, is crucial. This specifically includes interviewing and observing caregivers to better understand current stressors, family dynamics, and dyadic interactions between the pediatric patient and their caregivers (19).

Once the care team comprehensively understands the mental health needs of the pediatric patient and their family, appropriate intervention can be delivered. There are several evidence-based, trauma-informed relational interventions that are appropriate depending on the specific needs of the patient and their family. **Table 1** summarizes these approaches. These interventions can potentially be delivered during the child’s hospital stay and via telehealth post-discharge, which is an added benefit for young children who may be immunocompromised, live in rural areas, or face barriers to in-person follow-up (38, 46). Delivering early intervention via telehealth may also simplify logistics in care delivery and attenuate both caregiver and child stress. Moreover, equitable delivery of care that is proportionately targeted to needs would be in keeping with principles of social justice and is a cost-effective investment (31, 36).

**Table 1:** Evidence-based trauma informed relational interventions overview.

Intervention name	Target population	Modality of intervention	Goals	Mechanism of change	Research outcome
Child-Parent Psychotherapy (CPP)	Children ages 0-5 years with history of trauma (47, 48).	In-person or telehealth (49, 50).	Strengthen relationship between child and caregivers and heal and grow after trauma/stress (47, 51).	Using psychoeducation and in-the-moment feedback, caregivers can conceptualize child’s experience with trauma and current behaviors (48).	Improve attachment relationships, reduction in symptoms associated with PTSD and other mental health diagnoses (51).
Attachment Biobehavioral Catchup (ABC)	Children ages 0-36 months with a history of early life stressors (52).	In-person or telehealth (52, 53).	Promote healthy child-caregiver attachment through nurturance, following the child’s lead with delight, and avoiding intrusive/frightening behaviors (43, 52).	Through in-the-moment commenting and psychoeducation, caregivers can strengthen relationship with child, co-regulate stress, and serve as safe and secure base (52).	Improvement in attachment (43, 52, 54), diurnal cortisol regulation (41, 52, 55), and expressive language improvement (56).

Table adapted from Dahl et al. (19).

## Conclusion

There is a paucity of literature related to the mental health needs of early childhood oncology patients in whom critical care or prolonged hospitalization has been required. It is clear that PICU hospitalization has the potential to create an environment that disrupts normative developmental processes, primarily by interfering with important caregiver-child relationships. This puts children at risk for mental health disorders, even at an early age, including neurodevelopmental disorders, post-traumatic stress disorder (PTSD), anxiety, depression, and mood disorders (19). A systematic, collaborative approach to assessment is imperative in order to understand the unique needs of each patient and their family and provide the appropriate evidence-based intervention. While early brain development makes young children particularly vulnerable to the impact of stress, it may also represent an opportunity for neuro-adaptability and improved downstream resilience with early recognition and targeted, supportive intervention.
